# Yam Bean (*Pachyrhizus erosus* L. Urban) Powder Improves Grass Carp Myofibrillar Protein Gel by Forming Disulfide Bonds, Hydrogen Bonds, and Proper Microstructure

**DOI:** 10.3390/foods12102072

**Published:** 2023-05-21

**Authors:** Geyao Song, Kun Yang, Di Wu, Weiwei Lu, Rongshuo Chi, Jing Ma, Weiqing Sun

**Affiliations:** College of Life Science, Yangtze University, Jingzhou 434023, China

**Keywords:** myofibrillar protein, yam bean powder, gel characteristics, microstructure, chemical force

## Abstract

This study aimed to analyze the impact of different additions (0–1.25%) of yam bean powder (YBP) on myofibrillar protein (MP) gel characteristics such as the structure, water-holding capacity (WHC), chemical interaction strength of grass carp MP, and texture. The results showed that the YBP exhibited a strong water absorption capacity and filled in the protein heat-induced polymerization gel network well, which enabled the gel network to capture and retain water effectively, resulting in MP gels containing YBP with excellent WHC and gel strength (0.75%). In addition, YBP induced the formation of hydrogen and disulfide bonds in proteins and inhibited the conversion of α-helices to β-sheets and β-turn structures, facilitating the formation of high-strength gel networks (*p* < 0.05). In conclusion, YBP can significantly improve the thermally induced gelling properties of grass carp MP. In particular, the addition of 0.75% YBP had the best effect in terms of filling the gel network of grass carp MP, resulting in the formation of a continuous and dense protein network, leading to the composite gel with the best WHC and texture.

## 1. Introduction

China is rich in freshwater resources, and 70% of the fish in people’s diets are freshwater species. As the leader among the four most common domestic fishes being farmed, large numbers of grass carp (*Ctenopharyngodon idella*) are harvested annually. Freshwater fish proteins have poor gelling ability, especially grass carp, which largely limits their application prospects [1]. The gel properties of myofibrillar protein (MP) can greatly influence the structural characteristics, caking properties, appearance, and yield of meat products [2,3]. Exogenous additives such as polysaccharides, calcium chloride, and polyphenols are necessary during processing to improve the quality of MP gels [4,5]. In particular, polysaccharides are often used to increase the gel strength and water-holding capacity (WHC) of the MP gel and to improve the appearance and taste of products [6,7]. Polysaccharides are commonly found in all kinds of food crops. Different sources have different physical and chemical properties. Studies have shown that starch, the main component of wheat grinding powder, can modify the rheological properties of sardine surimi gel without affecting the thermal transitions [8]. Cassava starch can affect the storage modulus, gel characteristics, and microstructure of fish MP [9].

The yam bean (*Pachyrhizus erosus* L. Urban) is a legume that is rich in polysaccharides. It is from Mexico and has been promoted all over the world for many years [10]. In China, yam bean has been widely cultivated in Hubei and other provinces. The tubers of yam bean are edible parts with high nutritional value that contain carbohydrates and protein as well as large quantities of ascorbic acid, riboflavin, folic acid, and other beneficial substances [11]. The starch content of yam bean accounts for approximately 83% of the dry matter and has a certain antioxidant capacity, which may lead to good processing potential [12]. According to previous reports on traditional Chinese medical science, the nutritional composition of the yam bean tuber gives it the functions of producing fluid, quenching thirst, cooling, removing heat, eliminating alcohol and poisons, fighting cold, and lowering blood pressure and blood lipids [13]. It is often eaten as a raw fruit by Chinese people; moreover, the yam bean tuber is fried or made into soup to give play to its sweet and juicy flavor and health care value. At present, the development and utilization of yam bean is limited, and processed products are rarely seen in the market. In addition, there is no current method of durable storage, so every year, during the mature yam bean season, the yam bean market is overstocked and the yam beans rot.

The objectives of the present study were to explore the effect of the yam bean powder (YBP) composition on the gel properties and elucidate the interaction of YBP/MP composites. The microstructure and spatial distribution of YBP, MP, and water were observed to explore the changes in the secondary structure and chemical force of grass carp MP in the environment. This work is expected to provide a theoretical basis for the development of healthy grass carp/yam bean surimi products to meet the needs of consumers.

## 2. Materials and Methods

### 2.1. Materials

Yam bean tubers and grass carp were purchased from a local market in Wuhan, Hubei, China. The tubers were bought in April and weighed 800 ± 50 g each. Each fish weighed 2.5 ± 0.5 kg and was stunned immediately by being hit on the head with a wooden club and then gutted by a fishmonger. All chemicals used were of analytical grade.

### 2.2. Extraction of Myofibrillar Protein (MP) from Grass Carp

After removing the head, each grass carp was cleaned three times. The white muscles on the dorsal side and abdomen were collected and stored at −80 °C for a maximum of 2 weeks. The extraction of MP from the grass carp followed a previous method [14] with appropriate modifications. The meat was thawed at 4 °C for 12 h, chopped into surimi, mixed with phosphate buffered solution 1 (PBS_1_, 0.1 mol/L NaCl, 10 mmol/L Na_2_HPO_4_, 10 mmol/L NaH_2_PO_4_, 2 mmol/L MgCl_2_, and 1 mmol/L EGTA (pH 7.0)) and homogenized using a homogenizer for 120 s at 10,000 rpm at 4 °C (FSH-II, Jiangsu Jintan Huanyu Science Instrument Factory, Changzhou, China). Afterward, the turbid liquid was centrifuged at 2500× *g* at 4 °C for 15 min (Allegra X-30R Centrifuge, Beckman Coulter, CA, USA). The obtained precipitate was homogenized and centrifuged twice using the same method. After finishing the operation, three more homogenization–centrifugation cycles were performed while only replacing the PBS_1_ with sodium chloride solution (0.1 mol/L NaCl). The resulting precipitate, called MP, was collected and stored in a refrigerator at 4 °C. The MP concentration was measured using the biuret method, using bovine albumin as the standard.

### 2.3. Extraction of Dry Matter from Yam Bean into Powder

The yam bean powder (YBP) was isolated from the yam bean tubers and modified using a wet-milling procedure [10]. The tubers were crushed and liquefied with deionized water. The mixture was drained with double-layer gauze to separate the solids inside the gauze (mainly insoluble dietary fiber) [15], and the resulting solution was refrigerated at 4 °C for 24 h. After 24 h of rest, the solution was stratified to a supernatant and precipitate. After removing the supernatant, the precipitate was washed thoroughly with deionized water in order to remove the soluble compounds. This process was repeated three times. The final YBP was precipitated. Then, the water was poured out. The bottom layer was washed with ethanol for deodorization and dried at −80 °C for 48 h (Labconco FreeZone, Beijing Zhongke keer Instrument Company Limited, Beijing, China). Finally, the YBP was milled and sieved through #100 mesh (150 µm) and stored in a bag in a cool, dry place until it was used.

### 2.4. Preparation of MP/YBP Composite Gel

The MP was dissolved to its final concentration (30 mg/mL (*w*/*v*)) with PBS_2_ (0.6 M NaCl and 20 mM Na_2_HPO_4_/NaH_2_PO_4_ (pH 6.0)). Then, various fractions of YBP/MP solution (0.5%, 0.75%, 1.0%, and 1.25% (*w*/*w)*) were added. The sample without YBP was used as a control. The liquid mixture was stirred using a homogenizer for 5 min and centrifuged at 4 °C at 1200× *g* for 2 min to remove the bubbles.

The MP/YBP gel was prepared by injecting the mixture into a centrifuge tube, sealing the casing, and then heating the tube in a water bath using a two-step procedure (i.e., at 40 °C for 60 min and then at 90 °C for 30 min) [16]. After soaking the tube containing gel in an ice water mixture for 10 min to cool down, the resulting cylindrical composite gel was stored at 4 °C overnight for future analyses.

### 2.5. Water-Holding Capacity (WHC) of Complex Gel

The WHC was measured according to a previously described method [17]. Briefly, the gel was centrifuged at 12,000× *g* at 4 °C for 10 min to remove the water while recording the weight change at each step. The WHC (%, *w*/*w*) was expressed as grams of water held per 100 g of MP gel using the following equation:

$$\mathrm{WHC}\;\left(\%\right)=\frac{W_{2}-W_{0}}{W_{1}-W_{0}}\times 100$$

where *W*_0_ is the weight of the centrifuge tube; *W*_1_ is the weight of the tube with the gel and *W*_2_ is the weight of the tube plus the gel after the centrifugal water and the residual water on the surface of the gel have been aspirated with filter paper.

### 2.6. Textural Profile Analysis (TPA) of Complex Gel

A textural profile analysis (TPA) of the MP/YBP gel was performed with a texture analyzer (TA-XT Plus, Stable Micro Systems Ltd., Godalming, UK) using a previously described method [18] (described below) to measure the gel strength. The gel was cut into short cylinders of the same size and placed under the probe one at a time and then compressed at a compression degree of 80%. Both the pre-test and test speeds were set to 1 mm/s, and the post-test speed was set to 5 mm/s. The trigger force was set to 0.001 N, and the P/36R probe (diameter: 36 mm) was equipped.

### 2.7. Scanning Electron Microscopy (SEM) of Complex Gel

The procedures for the SEM analyses of the microstructures of the gels have been previously reported [19]. Briefly, the gel samples were cut into 1 mm thick slices, fixed with 2.5% (*w*/*v*) glutaraldehyde for 12 h, dehydrated (3 min) in gradient ethanol (50, 70, 80, 90, and 100% (*v*/*v*)), and freeze-dried for 48 h (Labconco FreeZone, Beijing Zhongke keer Instrument Company Limited, Beijing, China). The samples were platinum-sputtered for 1 min using an iron sputter coater and observed using an SEM instrument (JSM-IT 300, JEOL Ltd., Tokyo, Japan) working at an accelerating voltage of 15 kV.

### 2.8. Light Microscopy Observation of MP/YBP Composite Gel Double-Stained with PAS (Periodic Acid-Schiff Stain)/Naphthol Yellow S

Using a light microscope and PAS/naphthol yellow S double staining, the distribution and morphology of YBP in MP gel could be analyzed as described in previous report [20], with slight modifications. Specifically, the MP/YBP composite gel was fixed in a 10% (*v*/*v*) formalin solution and then dehydrated separately with gradient ethanol solutions (30, 50, 70, 80, 90 and 100%, *v/v*) and xylene, embedded in paraffin, and finally sliced, deparaffinized, and stained. The distribution of MP and YBP in the samples was observed using a light microscope (Eclipse Ci-L, Nikon, Tokyo, Japan). The staining was purple for YBP and yellow for MP.

### 2.9. Chemical Force of Complex Gel

The composite gel’s measurement was determined as stated in the report by Liu et al. [21]. Different forces can be broken by different chemical reagents such as urea and β-mercaptoethanol with different concentrations. A protein sample (2 g) was mixed with 15 mL of solution 1 (S_1_, 0.6 mol/L NaCl), homogenized with a homogenizer at 10,000 rpm for 2 min, and oscillated with a vortex mixer at 4 °C for 1 h (BR-2000, Bio-Rad Laboratories, CA, USA). The protein was allowed to fully dissolve and was then centrifuged at 10,000× *g* at 4 °C for 25 min. The supernatant was stored at 4 °C. Then, 15 mL of solution 2 (S_2_, 1.5 mol/L urea + 0.6 mol/L NaCl) was added to the precipitate, and the same procedure was carried out as described above. Then, the obtained precipitate was added to 15 mL of solution 3 (S_3_, 8 mol/L urea + 0.6 mol/L NaCl), and the process was the same as above. The precipitate was then mixed with 15 mL of solution 4 (S_4_, 0.5 mol/L β-mercaptoethanol + 0.6 mol/L NaCl + 8 mol/L urea) while the other operations remained unchanged, and the extracted precipitate was added to 2 mL of NaOH (1 mol/L) and stored at 4 °C. The supernatant obtained by centrifugation at each step was added to an equal volume of 20% trichloroacetic acid and then centrifuged at 5500× *g* at 4 °C for 15 min. The resulting precipitate was added to 2 mL of NaOH (1 mol/L) and stored at 4 °C. The protein concentration was detected using the Biuret method, and absorbance was measured at 540 nm. We divided each absorbance by the sum of the five absorbances of a group to find the relative content, which was the protein solubility. The difference between the gel solubility in S_1_, S_2_, S_3_, and S_4_ could be explained by the contributions from ionic bonds, hydrogen bonds, hydrophobic interactions, and disulfide bonds. The portion that could not be dissolved in S_4_ was considered to be the contribution of the non-disulfide covalent bonds [21].

### 2.10. Fourier Transform Infrared Spectroscopy (FTIR) of Complex Gel

The method was implemented according to a previous report [22]. The gels were dried at −80 °C for 48 h and then the dried samples were completely ground with potassium bromide (1/100 *w*/*w*), and the FTIR spectra were recorded using a FTIR spectrophotometer (IR-960, Tianjin Ruian Technology Ltd., Tianjin, China). The test parameters were a scan range of 4000–400 cm^−1^ and a resolution of 4 cm^−1^, and the scan was accumulated 64 times. Each group was scanned three times.

### 2.11. Low-Field Nuclear Magnetic Resonance (LF-NMR) of Complex Gel

A LF-NMR relaxation curve of the MP/YBP gel was recorded using a previous description by Han [23]. First, a sample (5 g) was placed into a cylindrical glass tube (15 mm in diameter) and inserted into a NMR probe. Then, a Niumag Benchtop (PQ001, Niumag Electric Co., Shanghai, China) was operated at 22.6 MHz at 32 °C. The spin–spin relaxation time (T_2_) was measured within a 90°–180° pulse sequence, with a scan interval of 6000 ms, 32 scans, and 8000 echoes, using the Carr–Purcell–Meiboom–Gill (CPMG) technique. The multiple exponential model was fitted using Multi Exp Inv analysis software Ver 4.0 (Niumag Electric Co., Shanghai, China).

### 2.12. Magnetic Resonance Imaging (MRI) of Complex Gel

MRI tests followed a previous method with a slight modification using a magnetic resonance imager (PQ001, Niumag Electric Co., Shanghai, China) [24]. By scanning the gel at heights of 1.0, 1.5, and 2.0 cm, proton density images of the gel were obtained. Then, the water distribution was visualized using pseudo-color maps derived from these images.

### 2.13. Statistical Analysis

All tests were replicated at least once using a new batch of MP. The data were processed using SPSS software Version 19 (SPSS Inc., Chicago, IL, USA) for statistical significance analysis using the one-way ANOVA function, and significant differences were defined at the *p* < 0.05 level using Duncan’s multiple range tests. The data were plotted using Origin 9.0 (v9.0, Origin-Lab, Massachusetts, USA) and the results are presented as the means ± standard deviations (SDs).

## 3. Results and Discussion

### 3.1. WHC and Gel Strength of MP/YBP Composite Gel

**Figure 1:**
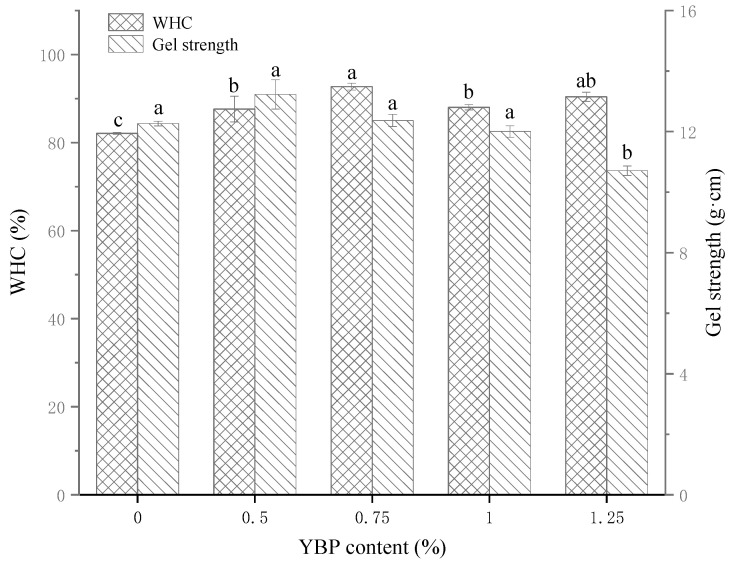
The WHC and gel strengths of MP gels with different amounts of YBP. The different letters (a–c) indicate significant differences between different amounts of YBP (*p* < 0.05). The data are the means ± standard deviations (n = 3). WHC, water-holding capacity; MP, myofibrillar protein; YBP, yam bean powder.

WHC is commonly used to evaluate the quality and yield of products because it indicates the binding ability of proteins to water [25]. Figure 1 shows the WHC values of the control and composite samples. With an increase in the YBP content, the WHC value also increased gradually. When the amount of YBP reached 0.75%, the WHC value of the gel reached its maximum (*p* < 0.05). This value subsequently decreased, then tended to be stable. There were two main reasons for the phenomenon described above: first, YBP particles acted as fillers in the gel network to help the MP gel network retain water at the low addition level (0–0.75%), which was consistent with many studies [26], and second, YBP granules have an amazing ability to absorb water and expand, and they have a large number of hydrophilic groups, which means that they can bind to water better than MP [9]. In the heat-induced gel two-phase system of YBP and MP, more added YBP will compete for water from the MP and expand its structure [27]. Some of the water might migrate from the MP domain to the YBP instead of being lost. However, rapid growth of the YBP particle volume also causes great damage to the gel network dominated by MP, making it difficult to intercept water [28]. Once the addition amount of YBP crosses the limited scope (0.75–1.25%), the YBP particles might break the continuity of the protein gel, causing a reduction in the composition and integrity of the network structure and even water loss. This is discussed in the section on the microstructure. This antagonistic relationship between water interception/absorption and structural damage eventually leads to a gradual increase in the WHC at a low addition amount, a decrease at a high addition amount, and ultimately a transient dynamic equilibrium state (*p* < 0.05).

Gel strength can be measured with the TPA, and the results can be used as the basis for evaluating gel quality [29]. Gel strength usually decreases with an increase in the WHC due to an increase in the water content and the swell of the protein network [30]. Figure 1 shows the gel strength values of all samples. When the additive amount increased, the first two groups’ gel strength values increased, while those of the latter groups decreased. This result shows that an appropriate addition of YBP (0.5–1%) can improve the gel strength, while too much YBP leads to insignificant or even lower gel strength (*p* < 0.05). After the thermal induction of pure YBP, the gel was characterized by low elasticity, high brittleness, a tendency to collapse, and weak strength and stability [31]. When YBP is added to the MP, the competition for water and the interwrapping between them inhibits the gelatinization degree of the YBP particles, in combination with the filling effect [32] and structural damage described above, thus affecting the texture profile data and the sensory properties of the system.

### 3.2. Microstructure of MP/YBP Composite Gel and Spatial Distribution of YBP in Composite Gel

#### 3.2.1. Network Structure

**Figure 2:**
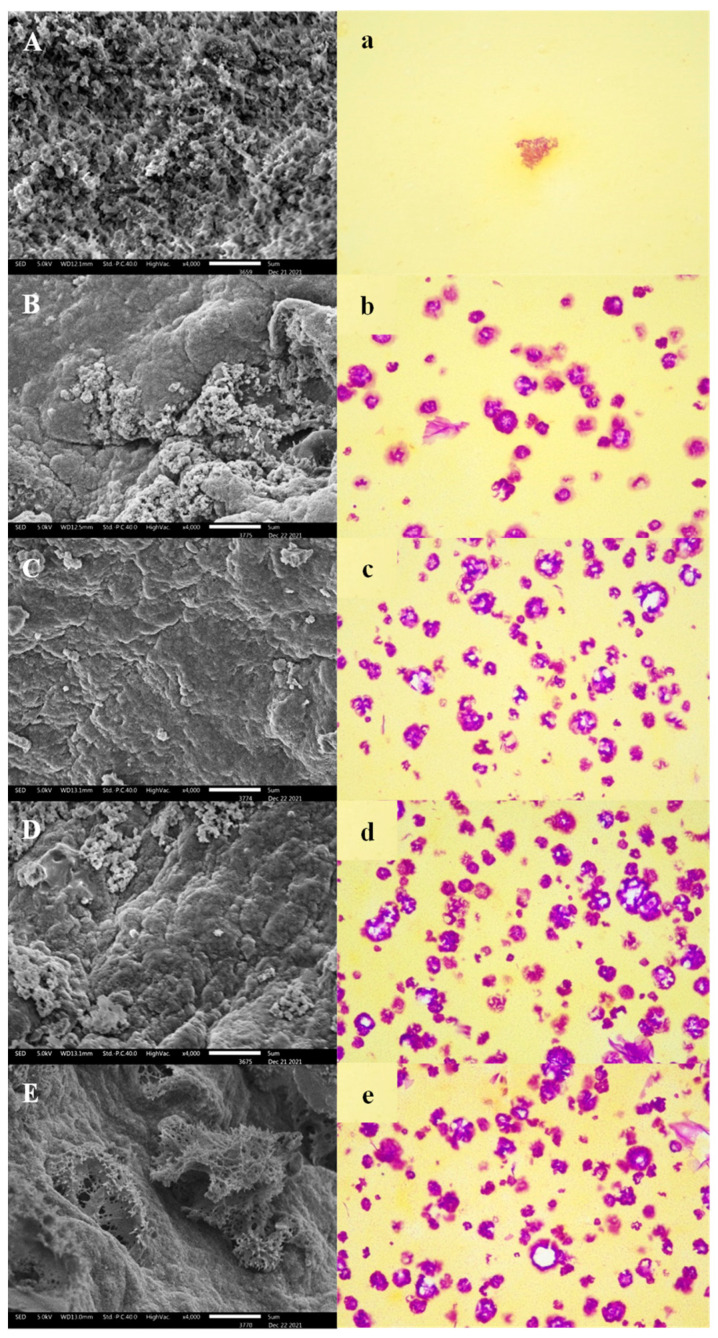
The gel microstructure of the MP gel, observed using a scanning electron microscope (4000× (**A**–**E**)), and light micrographs (400× (**a**–**e**)) with double staining. The staining was purple for YBP and yellow for MP. (**A**–**E**,**a**–**e**) represents the YBP levels of 0, 0.5, 0.75, 1, and 1.25%, respectively. MP, myofibrillar protein; YBP, yam bean powder.

The SEM photographs of the gels reflected their microstructure (Figure 2A–E), which was closely related to the WHC and the gel characteristics [33]. As the YBP content increased, the rough rolling surface of the gel was gradually covered by gelatinized YBP. At the same time, populated network channels appeared, and a relatively flat surface state formed (Figure 2C). However, by further increasing the YBP content, the steady state of the gel was destroyed, and the expansion of YBP squeezed or even penetrated the gel network (Figure 2E).

A pure MP network with large pores was formed by extruding water during heat induction [34]. The addition of YBP can facilitate this phenomenon by absorbing excess water and fixing the loose three-dimensional network, and its content and degree of gelatinization can influence the final result. The phenomenon reflected in this set of photos corresponds to the results of the WHC and TPA, which showed that an appropriate addition amount (0.5–1%) can make YBP granule gelatinization relatively sufficient, and that YBP can assist in supporting the whole system by absorbing excess water. The mass proportion and volume expansion degree in the gel do not destroy the integrity of the network and allow full play for the positive effect of the additives. An excessive addition amount is counterproductive as YBP does not only influence water interception but also breaks down the network structure to form new water channels, leading to water loss.

#### 3.2.2. YBP Distribution

It is generally believed that the effect of starch on MP gel is closely related to its gel properties, especially the high capacity for water absorption and volume expansion [32,35]. Therefore, the PAS/naphthol yellow S double-staining method can help us to observe the state of YBP in a heat-induced gel system (Figure 2a–e). The increase in the YBP content in the system is clearly reflected in Figure 2a–e. Fan et al. [33] reported that this kind of thermo-induced gel is actually a phase separation system in which starch is trapped in the primary gel network of MP as a secondary gel network due to the thermodynamic incompatibility of MP and starch. The MP/YBP composite gel formed a two-phase system that consisted of a separated YBP phase (purple) in a continuous MP matrix (yellow), and the control only had MP (all yellow filled) (in Figure 2a, the central purple color is PAS, not YBP). Surrounded by the MP network, the YBP in almost all samples showed an intact ellipse, probably because the spatial boundary and the available water limited its swelling. As shown in Figure 2b–e, the purple color existed both inside the YBP granules and on their periphery. The former may have been high-molecular-weight amylose that could not dissociate from the granules when heated. The latter was probably due to the dissociation of amylopectin and low-molecular-weight amylose [33].

In addition, the white color inside the granules may indicate that the YBP molecules migrated from the center to the periphery [28]. This color information indicates the degree of gelatinization of the YBP. It was observed that the gelatinization degree of the YBP did not change linearly with the increase in the YBP amount. When the addition amount was low, the YBP particles could not contend with the binding of the gel network; when the amount was too high, the available water was limited, and the gelatinization could not be completely ensured. YBP reached a relatively high gelatinization degree at a moderate amount (Figure 2c,d). At the same time, the WHC and TPA of the gel were also in the best state, which may have resulted in the better two-phase penetration [36] of YBP and MP.

### 3.3. Chemical Force of MP/YBP Composite Gel

**Figure 3:**
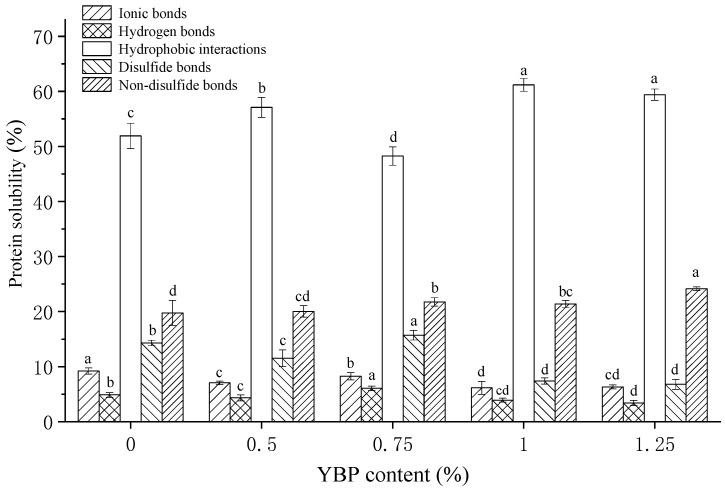
Chemical forces of MP gels with different amounts of YBP. The different letters (a–d) indicate significant differences between different amounts of YBP (*p* < 0.05). The data are the means ± standard deviations (n = 3). MP, myofibrillar protein; YBP, yam bean powder.

During the formation of heat-induced MP gels, the interactions between MP molecules mainly include chemical forces such as ionic bonds, hydrogen bonds, and hydrophobic interactions [37]. Figure 3 shows the relative contents of ionic bonds, hydrogen bonds, hydrophobic interactions, disulfide bonds, and non-disulfide covalent bonds. It is consistent that hydrophobic interactions were the dominant intermolecular force between MP molecules, rather than hydrogen bonds or ionic bonds [38]. With the addition of YBP, the contents of the ionic bonds, hydrogen bonds, and disulfide bonds decreased, while the contents of the hydrophobic interactions increased. However, when the YBP was 0.75%, all of the above changes were carried out in the opposite direction (*p* < 0.05). In addition, the non-disulfide covalent bonds increased gradually with the increase in YBP (*p* < 0.05).

MP is composed of actin, tropomyosin, troponins, and other low-molecular-weight proteins [39]. It is reported that added starch can interfere with some non-specific associations of these proteins in MP [40], which can explain the phenomenon that when the YBP levels increased in the pure MP gel, some of the interactions such as ionic bonds, hydrophobic interactions, and disulfide bonds decreased. However, the addition of YBP enhanced the protein–protein interactions at 0.75% YBP content, leading to increases in hydrogen bonds and disulfide bonds [40]. By changing the continuity, squeezing the MPs in the network, and competing for water with MP as a hydrocolloid, the presence of YBP might change the five chemical forces, as previously mentioned. These forces in the gel had different trends when appropriate amounts of YBP (0.75%) were added. This addition amount was the same as for the other detection indicators discussed earlier.

### 3.4. Molecular Structures of MP/YBP Composite Gel

**Figure 4:**
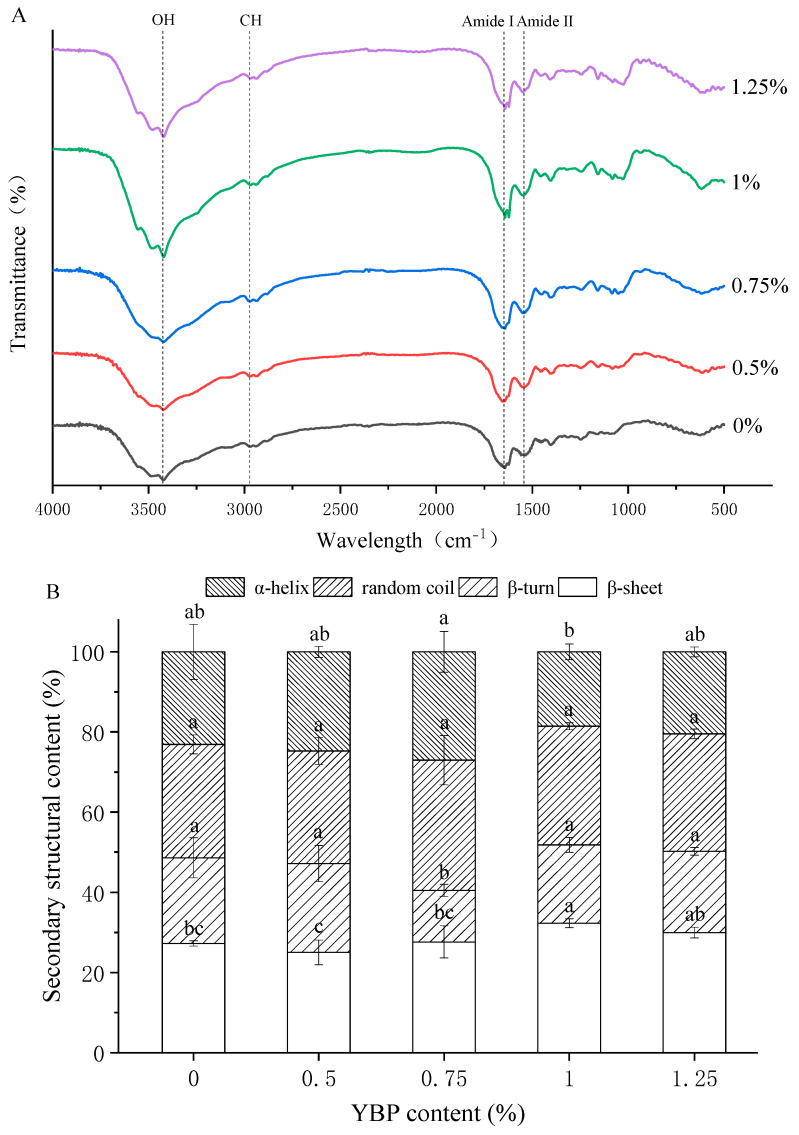
(**A**) FTIR spectra of the MP gels with different amounts of YBP. (**B**) The relative contents of various MP secondary structures in the MP/YBP composites with different amounts of YBP. The different letters (a–c) indicate significant differences between different amounts of YBP (*p* < 0.05). The data are the means ± standard deviations (n = 3). FTIR, Fourier transform infrared; MP, myofibrillar protein; YBP, yam bean powder.

Figure 4A shows the FTIR spectra of the blank and composite gels. Studies have shown that due to the stretching of the glucose ring, O–H, and C–H, pure starch has peaks at 1639 cm^−1^, 3438 cm^−1^, and 2931 cm^−1^ [41]. The protein also contains O–H and C–H, so the gel system shown in the figure had distinct peaks at 3438 cm^−1^ and 2931 cm^−1^. As a “fingerprint” for starch, the bands in the 800–1200 cm^−1^ range were dominated by ring vibrations that were overlapped by C–OH, C–C, and C–H side-group vibrations and the C–O–C glycosidic bond vibration [42]. Amide band I (1600–1700 cm^−1^) and amide band II (1500–1600 cm^−1^) are the characteristic peaks of the protein [43], and could also be observed in the FTIR spectra. Clearly, the peaks shown in Figure 4A were from MP or YBP. No other covalent bond peaks appeared, indicating there was no chemical interaction between them during heating.

According to previous studies, amide band I (1600–1700 cm^−1^) and amide band II (1500–1600 cm^−1^) are commonly used to monitor the secondary structure conformation of MP under different conditions. Among them, amide band I is considered to be the most reliable index of the protein secondary structure [44]. Amide band I can be divided into four bands, representing the secondary structures of protein that are known as α-helices (1650–1660 cm^−1^), β-sheets (1600–1640 cm^−1^), β-turns (1660–1700 cm^−1^), and random coils (1640–1650 cm^−1^). Figure 4B summarizes the relative areas of the bands, fit to the Fourier-deconvoluted spectra of the gel, to obtain the relative contents of the four structures. This shows that the appropriate addition (0.75%) of YBP can increase the content of α-helices and reduce the contents of β-sheets and β-turns (*p* < 0.05), but the secondary structures did not evidently distinguish themselves from others as the YBP increased. The result verified that the YBP molecules can protect MP against the transition from α-helices to β-sheets, which could ensure the gel properties. However, this is not concentration-dependent, which is consistent with previous reports [26,45].

### 3.5. Measuring Water Distribution of MP/YBP Composite Gel Using LF-NMR and MRI

**Figure 5:**
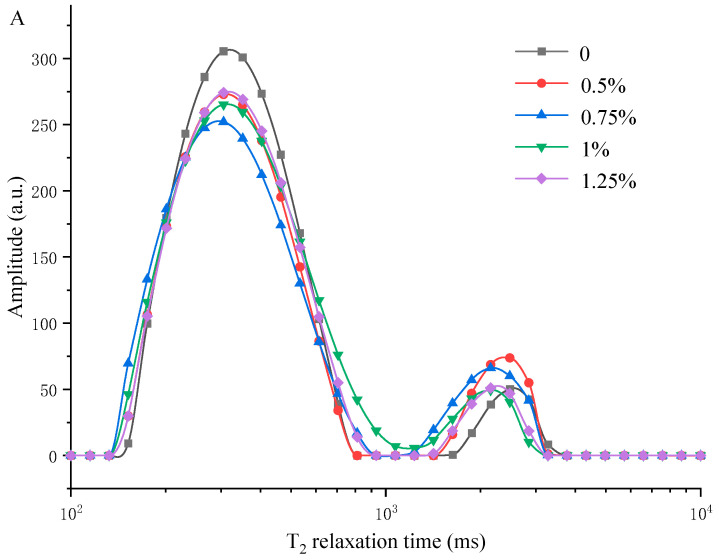

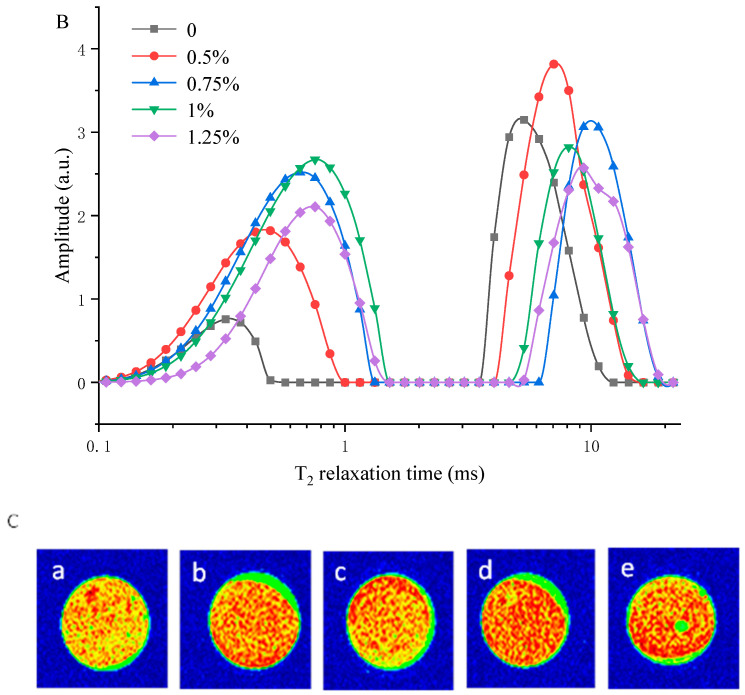
Water distributions of the MP gels with different amounts of YBP. (**A**,**B**) Curves of the T2 relaxation time. (**C**) Calorific value maps of moisture. (**a**–**e**): MP gels with 0, 0.5, 0.75, 1, and 1.25% YBP. MP, myofibrillar protein; YBP, yam bean powder.

T_21_, T_22_, and T_23_ are the relaxation times of bound water, immobile water, and free water, respectively, which can reflect the mobility of different water fractions in a MP gel system [34,46]. In Figure 5A,B, four peaks at 0.1–3 ms (T_21_), 3–13 ms (T_22a_), 70–700 ms (T_22b_), and 1000–4000 ms (T_23_) could be seen in all samples, and the immobile water (T_22a_ and T_22b_) was the predominant water component (>85%). The peaks of T_21_ and T_22a_ shifted in the direction of high relaxation time when the amount of YBP was appropriate (0.75–1%). Once the YBP level crossed 1%, the changes in T_21_ and T_22a_ would stop and even reverse course. Similarly, the value of T_22b_ decreased, but T_23_ increased under 0.75–1% YBP (*p* < 0.05), and both of them changed their trends after more YBP was added. This phenomenon of the opposite trends before and after the appropriate amount of YBP is consistent with the WHC, gel strength, and others. More specific proportions of the peak areas of the MP gels with different amounts of YBP are shown in Table 1. PT_21_ and PT_23_ increased with the increasing YBP level and peaked at 0.75% YPB content, but PT_22b_ decreased and peaked at 0.75–1%, and PT_22a_ has no significant change (*p* > 0.05). According to the aforementioned information, the bound water and free water in a composite gel increase when the amount of YBP increases, indicating that YBP has a good ability to restrict the free movement of water molecules [40], which makes it a powerful water absorbent. Although YBP changed immobile water into free water, this extra free water could also be retained in the system and did not affect the WHC because of the strong water absorption capacity of YBP. Moreover, the content of these three types of water were relative, with 0.75% having the best WHC, where the amount of free water in the system increased, and the relative amount of other kinds of water decreased.

The pseudo-color maps showed significant differences in the water distribution between the blank gel and the composite gels (Figure 5C). The more hydrogen protons a gel has, the more obvious the red color will be in the pseudo-color images [24]. The samples with the YBP appeared to contain more red areas than the control samples, which proves that the addition of YBP can effectively lock water in a MP gel system. It is worth noting that water was not evenly distributed in the high-YBP-content gel, which may have been another reason for the degradation of gel quality.

## 4. Conclusions

The characteristics of the heat-induced MP gel were significantly changed by the addition of YBP. With increases in YBP (0.5–1.25%), the WHC of the gel could increase by 5–12%, which was mainly attributed to the strong water absorption capacity of the YBP and its filling effect on the MP gel network. A moderate addition of YBP (0.5–0.75%) could improve the gel strength of the heat-induced MP gel. Specifically, 0.75% YBP had the most uniform and dense microstructure, and the YBP embedded in the network could also provide relatively sufficient gelatinization. The filling of the MP network’s water channels with YBP particles increased the network density and enhanced the interactions between MP molecules, thus forming a chemical bond environment high in disulfide bonds and low in hydrophobic bonds (*p* < 0.05). This also inhibited the transformation from α-helices to β-sheets. These changes corresponded to a MP gel with good gel strength and WHC. In conclusion, within the tested range, when the addition amount of YBP was 0.75%, its water absorption and expansion, filling effect on heat-induced MP gel, and changes in the continuity and compactness of the MP network reached a good balance, which made the composite gel with the best quality.

## Figures and Tables

**Table 1:** Proportions of the peak areas of MP gels with different amounts of YBP.

YBP Content (%)	PT_21_ (%)	PT_22a_ (%)	PT_22b_ (%)	PT_23_ (%)
0	0.40 ± 0.32 ^b^	0.59 ± 0.08 ^a^	92.47 ± 0.25 ^a^	6.54 ± 0.02 ^b^
0.5	0.58 ± 0.14 ^ab^	0.62 ± 0.15 ^a^	89.28 ± 1.80 ^ab^	9.52 ± 1.64 ^ab^
0.75	0.83 ± 0.21 ^a^	0.58 ± 0.14 ^a^	86.49 ± 3.62 ^b^	12.09 ± 3.57 ^a^
1	0.66 ± 0.31 ^ab^	0.71 ± 0.16 ^a^	87.24 ± 4.21 ^b^	11.39 ± 4.36 ^ab^
1.25	0.47 ± 0.19 ^ab^	0.66 ± 0.18 ^a^	89.19 ± 2.44 ^ab^	9.68 ± 2.48 ^ab^

Mean values with different lowercase letters (a, b) in each row differ significantly (*p* < 0.05). PT_21_, PT_22_, and PT_23_ are the percentages of bound water, immobilized water, and free water, respectively. MP, myofibrillar protein; YBP, yam bean powder.

## Data Availability

Data are contained within the article.
